# Organic matter from benthic foraminifera *(Ammonia beccarii)* shells by FT-IR spectroscopy: A study on Tupilipalem, South east coast of India

**DOI:** 10.1016/j.mex.2017.01.001

**Published:** 2017-01-10

**Authors:** G. Sreenivasulu, N. Jayaraju, B.C. Sundara Raja Reddy, T. Lakshmi Prasad, K. Nagalakshmi, B. Lakshmanna

**Affiliations:** a Department of Geology, Yogi Vemana University, Kadapa, Andhra Pradesh, India; b Department of Geology, Sri Venkateswara University, Tirupati, Andhra Pradesh, India; c Department of Earth Sciences, Yogi Vemana University, Andhra Pradesh, India

**Keywords:** FT-IR Spectroscopy procedure was used as mentioned by Ref # 15, Benthic foraminifera, Organic matter, FTIR spectrophotometer, Biodegradation

## Abstract

Fourier Transform Infrared Spectroscopy (FTIR) was used to study the variations in organic matters of benthic foraminifera *(Ammonia beccarii)* from four samples collected from beach environments from brackish environments along Tupilipalem coast (South east coast of India). Common absorption bands were observed as peaks in the range of 3600–3400 cm^−1^, 3000–2850 cm^−1^, 1750–1740 cm^−1^, 1640–1600 cm^−1^, 1450–1350 cm^−1^, 885–870 cm^−1^ and 725–675 cm^−1^ in all the shells of *Ammonia beccarii*. The FTIR spectrum of station-1 represents the presence of alkanes (CH_3_) and alkyl halide (C—F stretching) with absorptions at the range 1385–1255 and 1350–1150 cm^−1^ were observed and ether (C—O stretching) absorption band was observed at stations 1 and 3 with wavenumber of 1115 cm^−1^ and 1117 cm^−1^ respectively. Alkynes C—H bend was observed at station-1 with the wavenumber of 667.43 cm^−1^. The shifting of peak positions in all the samples is could be due to presence of organic matter in the samples. Satellite remote sensing and field observation data revealed that the river mouth at Tupilipalem coast was closed by a sand bar. Consequentially, this waterbody may affect the species diversity.

• Positions of the sampling locations were identified using a hand-held Garmin Global Positioning System (GPS).
• Foraminifera from the sediment were obtained using a mixture of Bromoform and Acetone.
• The functional groups present in the benthic foraminifera shells were recorded in the spectral range of 4000–400 cm^−1^ using an FT-IR Spectrophotometer.

## Graphical abstract

**Figure:**
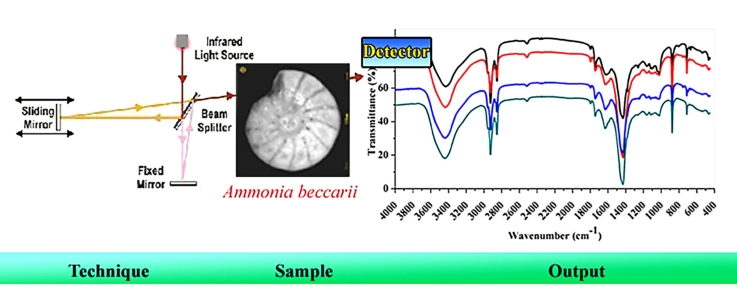

## Introduction

Foraminifera are single-celled organisms (protists) with a long-lasting geological record that span the Earth's entire Phanerozoic history (∼570 My) [1]. They are widely distributed in all marine environments. They play a significant role in global biogeochemical cycles of inorganic and organic compounds [2]. The fossil foraminiferal studies help to assess the palaeo-depth, palaeosalinity and palaeo-environment and are useful in palaeogeographic reconstruction and ecological studies [3]. Foraminifera are valuable indicators of past environmental change [4]. Their sensitivity to pollutants may be expressed by modification in test form or assemblage composition [5], [6]. Furthermore, many foraminiferal taxa secrete a carbonate indicating and evidence of environmental stresses through time. They are commonly small and abundant compared to other hard-shelled taxa and are easy to collect, providing a highly reliable database for statistical analysis, even when only small sample volumes are available [7]. The calcium carbonate shell in foraminifera is of interest because it varies not only with species but also with a variety of environmental factors such as temperature, pH, ionic composition. The CaCO_3_ structure probably affects such physical properties of the shell as appearance and shock resistance-of importance to commercial interests which use shells. Test of the foraminiferal fauna has organic components (linings and cement) of a muco-polysaccharide, or glycoprotein nature in which the basic bio-molecular structure has a proteinaceous part linked to an amino sugar/glycogen unit.

The application of advanced high resolution spectroscopic techniques in the investigation of organic test materials be a value addition to the previously acquired information. The mechanisms employed by the foraminifera using adhesive materials to aid, and form secure structures, in test wall construction, in aqueous environments, may provide specific information useful in wider applications. Additional information regarding the composition of the organic components (cements and linings) may enhance the established classification scheme. The overall strength of the organic materials and degradation to the resistance may be key for research in areas of material science and underwater technology [8]. The qualitative aspects of infrared spectroscopy are one of the most powerful attributes of this diverse and versatile analytical technique [9]. Fourier Transform Infra Red (FTIR) is considered to be the most preferred method of infrared spectroscopy. Where in, IR radiation is passed through a sample. The resulting spectrum represents the molecular absorption and transmission, creating a molecular fingerprint of the sample. The commonly used region for infrared absorption spectroscopy is 4000 ∼ 400 cm^−1^ as the absorption radiation of most organic compounds and inorganic ions is within this region.

## Study area

**Fig. 1:**
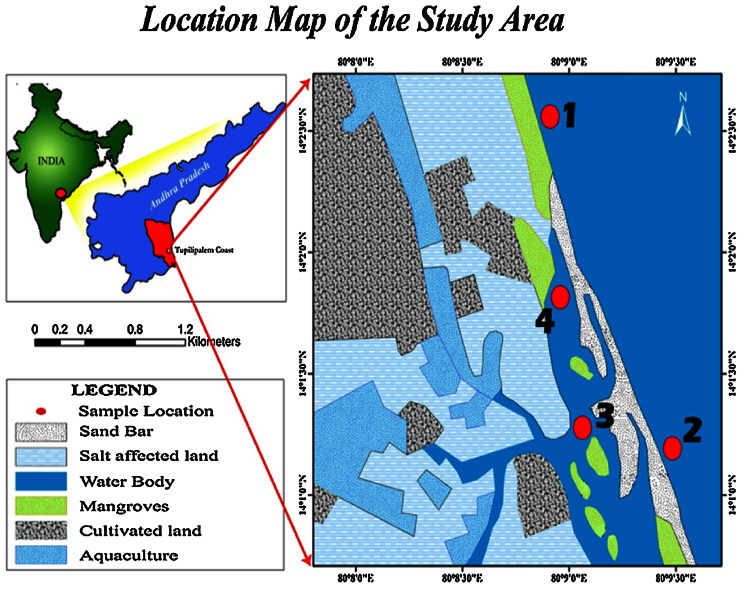
Location map of the study area.

Tupilipalem is 20 km away from Dugarajapatnam (southeast coast of India) and about 120 km away from Pulicat Lake and the satellite launching centre at Sriharikota. Tupilipalem is one of the proposal sites for constructing a major port to be named as Dugarajapatnam Port. The study area is geographically located in the southern part of Nellore district, lying between latitude 14°01′0″–14°02′30″N and longitude 80°08′0″– 80°09′30″E in Andhra Pradesh, India The study area has a cover of mangrove swamps. These mangrove swamps are major contributions to the load of organic matter and fine grained sediments. Average annual rainfall in the study area is 1041 mm and the average maximum and minimum temperatures are 39.6 and 20 °C respectively. However, the currents in the study area are also affected by the tidal cycles, by the action of waves, the shore line geography and also by the presence of different water masses, assuming predominantly a SW-NE direction. At the time of sampling, the river mouth was closed with a sand bar due to currents (Fig. 1).

## Methodology

Four bottom sediment samples were collected from four locations (two samples from brackish water stations 3 and 4) and another two from a beach environment (stations 1 and 2) of Tupilipalem Coast, Bay of Bengal, India, by fishing boat using manually pushed auger [10]. Positions of the sampling locations were identified using a hand-held Garmin Global Positioning System (GPS) [11], [12]. The samples thus obtained was air dried at ambient temperature. Nearly 100 g sample was obtained after cone and quarter. Samples were spilt using a micro splitter and benthic foraminifera were picked and identified. Quantitatively, foraminifera could not be separated completely by floating in carbon tetrachloride [11], [13]. Foraminifera from the sediment were best obtained using a mixture of Bromoform (specific gravity 2.8) and Acetone (specific gravity 2.4) [14].

The functional groups present in the benthic foraminifera shells were recorded in the spectral range of 4000–400 cm^−1^ using an FT-IR Spectrophotometer (Perkin–Elmer Spectrum-2, UK). Prior to the analysis, the waveguide chamber was purged with dry air for 5 min to minimize the water vapor interferences. The recording was performed with a resolution of 4 cm^−1^ and 16 scans per sample. Each spectrum was corrected against pure KBr and the ambient air as a background spectrum [15]. The resulting spectrum represents the molecular absorption and transmission, creating a molecular fingerprint of the sample.

## Results

**Fig. 2:**
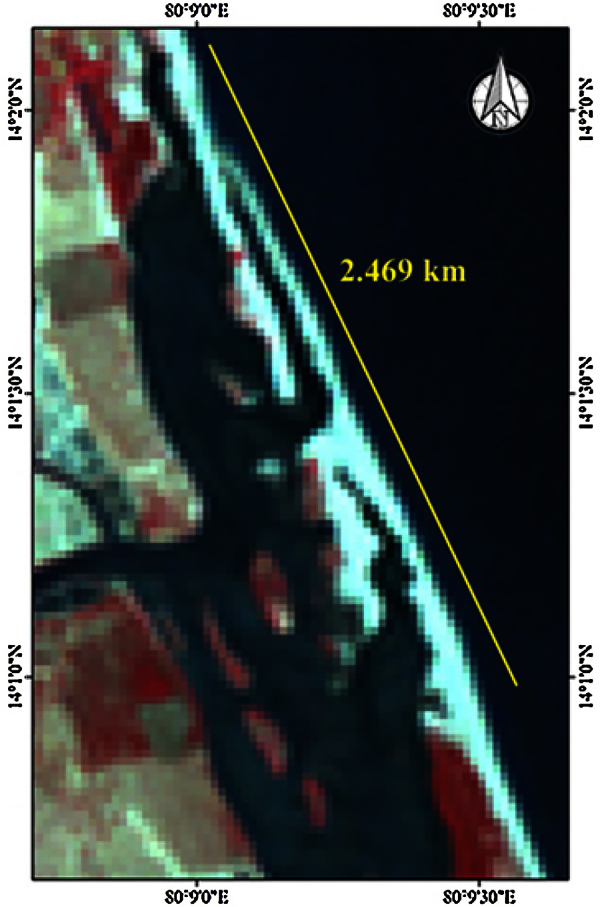
Satellite imagery showing the closed river mouth in the study area.

**Fig. 3:**
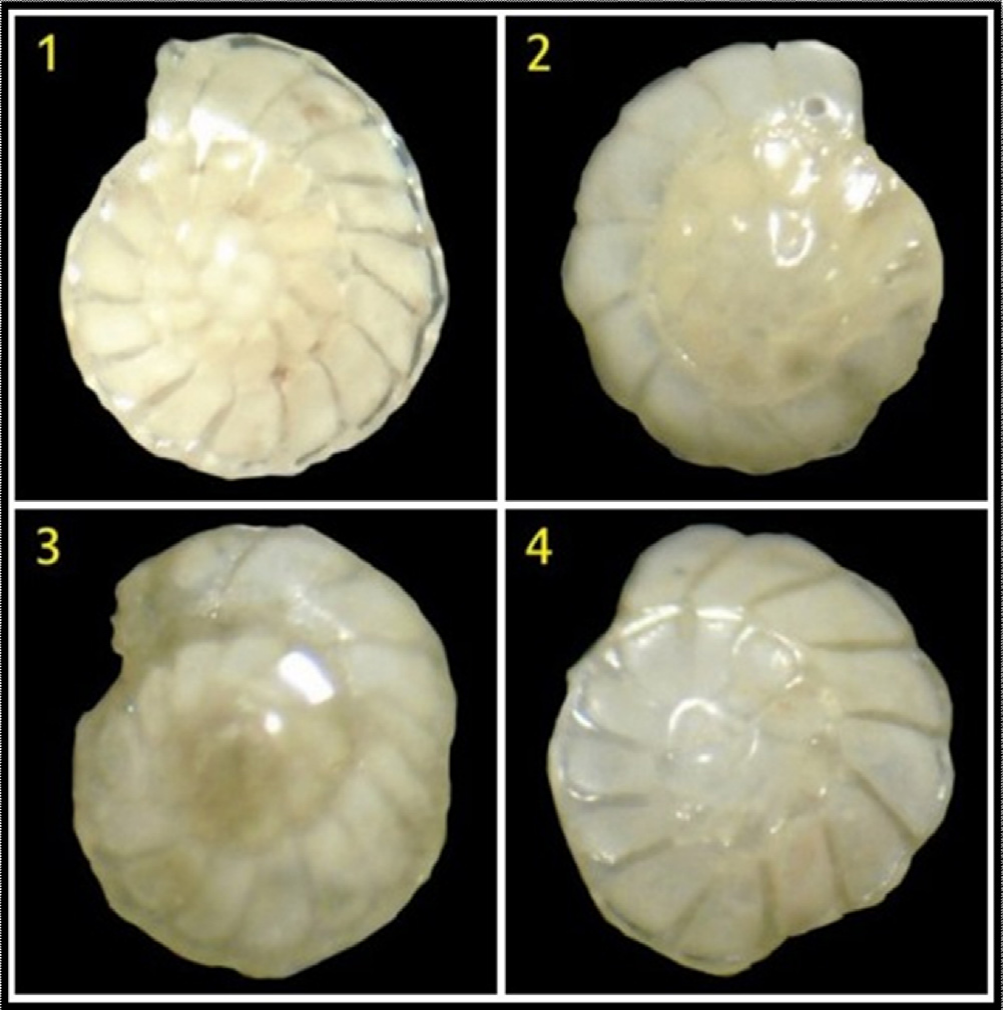
Benthic foraminifera *(Ammonia beccarii)* stations; 1&2 from beach environment and 3&4 from brackish environment.

**Fig. 4:**
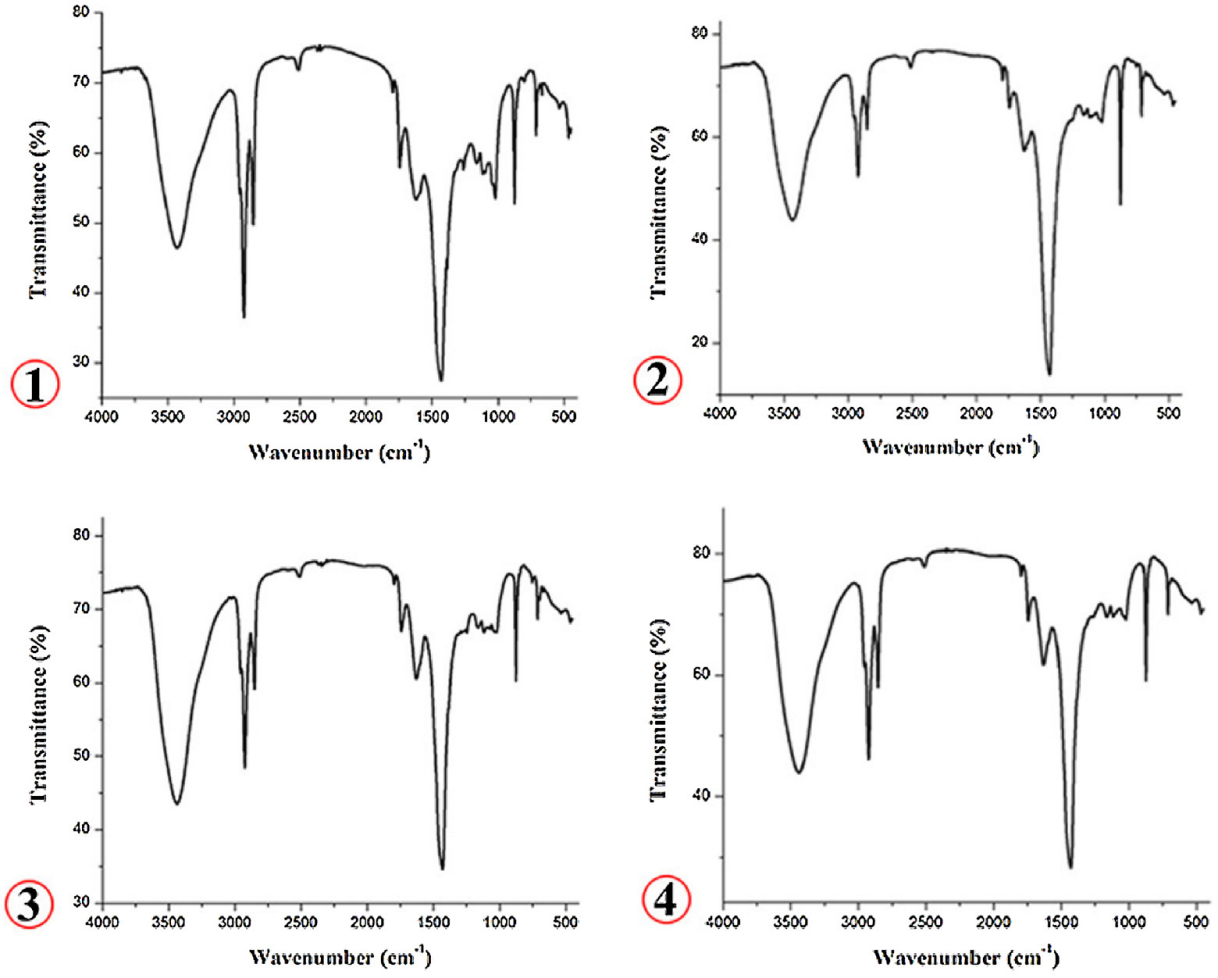
FTIR spectra of foraminiferal shells 1–4.

Four shells of benthic foraminifera *(Ammonia beccarii)* were analyzed to determine the variations in organic matter. Out of four, two shells were obtained from beach environments (stations 1 and 2) and remaining two (stations 3 and 4) from brackish environments of Tupilipalem coast where the river mouth was closed with a sand bar (Fig. 2). The IR spectra of all *Ammonia beccarii* (benthic foraminifera) shells show high absorption in the 4000 and 400 cm^−1^ wavenumber region (Fig. 3, Fig. 4) indicating prospective scope of research on organic compounds. These regions will be discussed individually and in future attempts will be made to relate absorptions to specific molecular vibrations.

### Beach environment

The beaches are sites, with human inhabitation and the beach ecosystems have often shown a high sensitivity to environmental change. These zones may be affected by a large series of anthropogenic-derived pressures, such as unbalanced inorganic nutrient input, that may cause anomalous development of primary production altering the structure of the trophic webs [16]. Benthic foraminifera *(Ammonia beccarii)* from stations 1 and 2 were selected to be analyse with FTIR spectroscopy to determine the organic matter. Two species were studied, and it was observed that there was a strong absorption band at 3431 and 3435 cm^−1^ at stations 1and 2 respecively, these bands show the presence of hydroxy groups and H-bonded OH stretching. The broad absorption bands at 2924.51 and 2853.85 cm^−1^ at station 1 and 2925 cm^−1^ and 2854 cm^−1^ at station 2, indicates the presence of alkanes (CH stretching) and alkanes which reflect the presence of fats and waxes [17]. A series of absorption bands at 1384.83 cm^−1^ and 1261.93 cm^−1^ were observed in fingerprint region revealing the presence of alkanes CH_3_ only at station 1. No alkanes absorption peaks at station 2 in the finger print region. Acidic Chloride C=O stretching absorption was observed at 1791.91 cm^−1^ and 1797.32 cm^−1^ at both stations 1 and 2 respectively. Absorption peaks at 1744.92 cm^−1^ and 1744.35 cm^−1^ indicates the presence of ketones C=O stretching at both stations 1 and 2. Amide N—H peaks observed at 1618.18 cm^−1^ and 1626.86 cm^−1^. At station 1, the presence of alkyl halides, C-F stretching was observed at 1161.19 cm^−1^ these alkyl halides tend to be toxic, they deter predator in marine organisms [18]. Wavenumber 1115.55 cm^−1^ peak was observed at station 1 and it indicating ether, C—O stretching. Aromatics have C—H out of plane absorptions at 875.80 cm^−1^ and 876.35 cm^−1^ at both stations 1 and 2 respectively. The concentrations of aromatic compounds might be the result of macromolecular thermal alteration. Alkenes, =CH out of plane has absorbed at a peaks of 712.36 cm^−1^ and 712.61 cm^−1^ for both the stations. At peaks wavenumber 667 cm^−1^ was observed displaying the presence of alkynes; C—H bending vibration at station 1.

### Brackish environment

Brackish water regions cause stress to the ecosystem because of their fluctuating environmental conditions. This stress reduces the species diversity [19]. Increasing of stress by adding polluting wastes to the brackish water, which is in vogue, may cause serious damage to ecosystem. The Brackish environment is different from open sea water for many reasons. For instance, the biological activity for brackish water is modified significantly due to its higher nutrient concentration. With this high amount of nutrients, fouling can be a serious problem. Bottom sediment samples from two locations from brackish environment were collected and processed for benthic foraminifera shells. Single benthic foraminifera shell was selected from each sample location. Two benthic foraminifera shells (stations 3 and 4) were analyzed with FTIR spectroscopy.

In the organic linings of calcareous foraminifera, a band in the region 3436.87 cm^−l^ and 3436.90 cm^−l^ arises from the stretching vibration of a hydroxy group, H-bonded OH stretching was observed at both station 3 and 4. Alkanes CH stretching was observed within the absorption range of 2925.02–2854.36 cm^−1^ at station 3 and 2924.90–2854.36 cm^−1^ at station-4. An absorption band at 1797.61 cm^−1^ was observed and it represents acid chloride C=O stretching at station 4. No acid chloride peak was observed at station 4. Ketones C=O stretching absorption band was observed at a peak range 1743.35 cm^−1^ and 1744 cm^−1^ at station 3 and 4 respectively. Absorption peak at 1630.49 cm^−1^ and 1633.71 cm^−1^indicates the presence of Amides; NH out of plane at stations 3 and 4. P = O phosphate was present at station 4 only at 1163.09 cm^−1^ wavenumber. There is no phosphate group at station 3. Ether; C—O stretching band was observed at station 3 at 1117.83 cm^−1^ wavenumber but it absent at station 4. The presence of aromatics were noticed at 876.07 cm^−1^ and 876.16 cm^−1^. In addition, C—H out of plane band was recognized. Alkenes =CH out of plane band was observed at peaks of 712.46 cm^−1^ and 712.52 cm^−1^.

## Discussions

The river mouth is important as sea water flows into the closed water body (river mouth) during high tides and river mouth water flows into the sea during low tides [20]. Above observations revealed that the river mouth was closed with sand bar formation. The river mouth retrospective data were retrieved by Satellite remote sensing where as direct observation data in the field. This may pose a threat to the biodiversity in closed water body (Fig. 2).

FTIR spectroscopy was used to study the variations of organic matter in foraminifera shell between either side of the sand bar (sea and closed water body) at Tupilipalem coast. The IR spectra of all benthic foraminifera shells show high absorption in the 4000 − 400 cm^−1^ wavenumber region (Table 1). A total of four benthic foraminifera shell belong to same genera collected from four stations. Out of four, two from sea side (beach environment) and another two from brackish environment (closed water body). Above results show the common absorption bands from 3500 to 1400 cm^−1^ region with significant shifting of frequencies. In finger print region (1400–400 cm^−1^) the alkanes CH_3_ absorptions at 1384.83 cm^−1^ and 1261.93 cm^−1^ were observed at station 1 from beach environment only. No alkanes CH_3_ bands were present at the remaining stations 2, 3 and 4 in finger print region. Alkile halides C—F stretching absorption band at wavenumber 1161.19 cm^−1^ was observed only at station 1. In the case of remaining stations, it was absent. Ether C—O stretching band was observed at stations 1 and 3 only at absorption band with wavenumbers 1115.55 and 1117.83 cm^−1^ respectively. Alkynes #C—H bend occured at station 1 only. No alkynes absorption peak was observed at stations 2, 3 and 4.

**Table 1:** FTIR frequency wavenumber and its assignment of benthic foraminifera.

S. No.	Frequency wavenumber (cm^−1^)				Assignment
	1	2	3	4	
1.	3431.27	3435.95	3436.87	3436.90	Hydroxy group, H-bonded OH stretch
2.	2924.51	2925.05	2925.02	2924.90	Alkanes; CH stretch
3.	2853.85	2854.36	2854.36	2854.36	Alkanes; CH stretch
4.	1791.91	1797.32	–	1797.61	Acid Chloride; C=O stretch
5.	1744.92	1744.35	1743.35	1744	Ketones; C=O stretch
6.	1618.18	1626.86	1630.49	1633.71	Amides; NH out of plane
7.	1430.74	1429.85	1431.50	1431.55	Misc. S=O sulfate ester
8.	1384.83	–	–	–	Alkanes; CH_3_
9.	1261.93	–	–	–	Alkanes; CH_3_
10.	1161.19	–	–	–	Alkyle halide; C—F strech
11.	–	–	–	1163.09	Misc. P=O phosphate
12.	1115.55	–	1117.83	–	Ether; C—O stretch
14.	875.80	876.35	876.07	876.16	Aromatics; C—H out of plane
15.	712.36	712.61	712.46	712.52	Alkenes; =CH out of plane
16.	667.43	–	–	–	Alkynes; #C—H bend

## Conclusions

In this study, an attempt has been made to quantify the organic matter using FTIR spectroscopy in benthic foraminifera shell from Tupilipalem coast, South east coast of India. FTIR spectra for all the four shells showed peaks associated with organic matter and carbonate. Results show high absorption in 4000–400 cm^−1^ region. Common peaks were identified within the spectrum range of 3500–1400 cm^−1^with significant shifting of frequencies due to organic substances in all the four locations. In most cases, both a shift to a higher frequency and an increase in absorption strength for the band were observed from beach and brackish environments. It indicates that the biological activity for brackish water is modified significantly due to its higher nutrient concentration. In finger print region (1400–400 cm^−1^) there is no Alkanes (CH_3_), Alkines (#C—H) overtones were observed from the shells of brackish environment. Satellite Remote Sensing and field observation data revealed that sea mouth at Tupilipalem coast was closed with sand bar. Above results show the common absorption bands from 3500 to 1400 cm^−1^ region with significant shifting of frequencies. In finger print region (1400–400 cm^−1^) the alkanes CH_3_ absorptions at 1384.83 cm^−1^ and 1261.93 cm^−1^ were observed at station 1 from beach environment only. No alkanes CH_3_ bands were present at the remaining stations 2, 3 and 4 in finger print region.
